# Universal routine HPV vaccination for young girls in Uganda: a review of opportunities and potential obstacles

**DOI:** 10.1186/1750-9378-7-24

**Published:** 2012-09-05

**Authors:** Cecily Banura, Florence M Mirembe, Anne R Katahoire, Proscovia B Namujju, Edward K Mbidde

**Affiliations:** 1 Child Health and Development Centre, Makerere University College of Health Sciences, P. O. Box 6717, Kampala, Uganda; 2 Department of Obstetrics and Gynaecology, Makerere University College of Health Sciences, P.O. Box 7072, Kampala, Uganda; 3 Uganda Virus Research Institute, P.O. Box 49, Entebbe, Uganda; 4 National Institute for Health and Welfare, Oulu, Finland

## Abstract

This article reviews the existing realities in Uganda to identify opportunities and potential obstacles of providing universal routine HPV vaccination to young adolescent girls. Cervical cancer is a public health priority in Uganda where it contributes to about 50–60% of all female malignancies. It is associated with a dismal 5-year relative survival of approximately 20%. With adequate financial resources, primary prevention through vaccination is feasible using existing education and health infrastructure. Cost-effectiveness studies show that at a cost of US$2 per dose, the current vaccines would be cost effective. With optimal (≥70%) coverage of the target population, the lifetime risk of cervical cancer could be reduced by >50%. Uganda fulfils 4 out of the 5 criteria set by the WHO for the introduction of routine HPV vaccination to young adolescent girls. The existing political commitment, community support for immunization and the favorable laws and policy environment all provide an opportunity that should not be missed to introduce this much needed vaccine to the young adolescent girls. However, sustainable financing by the government without external assistances remains a major obstacle. Also, the existing health delivery systems would require strengthening to cope with the delivery of HPV vaccine to a population that is normally not targeted for routine vaccination. Given the high incidence of cervical cancer and in the absence of a national screening program, universal HPV vaccination of Ugandan adolescent girls is critical for cervical cancer prevention.

## Introduction

Almost every case of cervical cancer is potentially preventable. Yet, women in low- as opposed to those in high-income settings have about a two fold cumulative risk of developing cervical cancer before the age of 65 years [1]. Equally, women in low income settings have a threefold risk of dying from cervical cancer than those in high income settings [2]. Human papillomavirus is the primary cause of cervical cancer (>99%) and is responsible for 83-95% anal, 87% oro-pharyngeal, 60-65% vaginal and 20-25% vulvar cancers in women [3]. A woman’s lifetime risk of acquiring HPV infection is greater than 80% and most infections occur within 3–4 years of sexual debut [4,5]. Among HIV positive women, the prevalence of HPV infections and high grade cervical pre-cancer lesions (CIN 2/3) is several fold higher than in HIV negative women [6,7]. Cervical cancer is the biggest single cause of years of lost life particularly in low income settings where it affects relatively young women at the peak of their productive years [8].

Preventive HPV vaccines that protect against HPV 16 and 18 have been commercially available since 2006. Cervarix® made by GlaxoSmithKline (GSK) Biologicals, Rixensart, Belgium and Gardasil® made by Merck & Co. Inc.,Whitestation, NJ, USA. The antigens in these vaccines protect against oncogenic HPV types 16 and 18 responsible for 70% of cervical cancers, globally [9]. The remaining 30% of cervical cancer cases are caused by other oncogenic HPV types. Results from recent studies have shown some level of cross protection by both vaccines [10]. This implies that screening programs will still be needed even after HPV vaccines are introduced. Gardasil also prevents non oncogenic HPV 6 and 11 responsible for about 90% of genital warts but rarely cause anogenital cancers [11].

The introduction of these two vaccines presents an opportunity to prevent approximately 70% of cervical cancer cases, globally [12]. Data from Sub Saharan Africa however, seem to suggest that other oncogenic HPV types are more prevalent and more diverse than elsewhere in the world possibly because of prevalent HIV infection [13,14]. This introduces uncertainty about the proportion of cervical cancer cases that will be potentially prevented by vaccination.

There is an urgent need for preventive HPV vaccines in low-income settings particularly in Sub Saharan Africa (SSA) given the elevated age standardized incidence rates of cervical cancer [15]. However, the demand for these vaccines so far is low. By the end of 2011, over 30 high- and middle- income settings had introduced routine HPV vaccination in their national vaccination programs compared to only one (Rwanda) in SSA [16]. Past experience has shown that there is usually a lag period of 15–20 years between the introduction of new vaccines in high- and low- income settings due to issues of financing, procurement and alternative pathways to vaccine development and production [17].

The World Health Organization (WHO) recommends that routine HPV vaccination of young adolescent girls could be integrated in national immunization programs if 5 key criteria are fulfilled [18]. These include (i) Prevention of cervical cancer and other HPV related-diseases, or both constitute a public health priority (ii) Vaccine introduction is programmatically feasible (iii) Sustainable financing can be secured (iv) The cost-effectiveness of vaccination strategies in the country or region is considered (v) HPV vaccination is targeted to adolescent girls prior to sexual debut. This article reviews the existing realities in Uganda to identify opportunities and potential obstacles of providing universal routine HPV vaccination for young adolescent girls.

## Current realities

### Prevention of cervical cancer and other HPV related-diseases, or both constitute a public health priority in Uganda

National data on cervical cancer burden in Uganda is not available. However, based on data from the population-based Kampala Cancer Registry (KCR), Uganda has one of the highest cervical cancer incidence rates in the world [19]. The age-standardized incidence rate per 100, 000 is 47.5, which is more than three times the global estimate (15.8) [20]. Cervical cancer is the most frequently diagnosed cancer among women and the 2^nd^ most frequent cancer among women aged 15–44 years. It contributes to about 40% of all malignancies reported by KCR, which represent 50-60% of all female malignancies. Current estimates show that annually, approximately 3500 women are newly diagnosed and 2400 die from cervical cancer. Projections show that by 2025, about 6400 new cervical cancer cases and 4300 deaths will occur annually [20].

Since the 1960s, studies have shown an overall trend of elevated cervical cancer incidence among Ugandan women [21], which are probably an underestimate since most women aged 15 years and older and at risk for cervical cancer do not access health care. Not all health facilities are equipped to provide a full range of health care services and referral services seldom work. There is no organized national screening and treatment program. The limited number of trained personnel, infrequent supply of basic materials, poorly equipped laboratory facilities and the potential difficulty of following-up women with positive smears in a setting where there is neither national identification cards nor street addresses make it difficult to have an organized national screening and treatment program [22].

Access to new proven and appropriate low cost screening technologies such as visual inspection with acetic acid or Lugol’s Iodine (VIA/VILLI) that are based on “screen and treat” of positive cases in a single visit are limited to only a few referral health facilities. Similarly, the new HPV-based screening technologies such as the *care*HPV™ test (Qiagen, Gaithersburg, MD, USA) presently under evaluation at the national referral hospital is accessed by very few women. Consequently, opportunistic cytology-based screening is offered upon request in a few selected hospitals.

The majority (72%) of cervical cancer patients seen at the national referral hospital present with advanced disease [23]. Most of these women have never heard of cervical cancer. Given the constraints posed by limited resources in the public health sector, the availability of care and treatment largely depend on the patient’s financial contribution. Home- based palliative care is limited in geographical coverage [24]. Consequently, the prognosis of cervical cancer patients is very poor with a 5-year relative survival of approximately 20% compared to about 64% for black Americans of USA [25].

Ugandan women are disproportionately affected by HIV/AIDS accounting for 57% percent of approximately 1.2 million adults living with HIV [26]. Many studies in Uganda have shown a higher prevalence and incidence of oncogenic HPV infections among HIV positive than HIV negative women [7]. Compared to HIV negative women, HIV positive women seem to have higher risk [27] and early onset of cervical cancer [28]. Although HIV positive women seem to have a higher risk of dying from cervical cancer, their 3-year relative survival is similar to that of HIV negative women [29].

Other HPV-related cancers including anal, vulvar, vaginal and oro pharyngeal (excluding nasopharynx) are rare in Ugandan women [20].

### Vaccine introduction is programmatically feasible

The Uganda National Drug Authority registered both HPV vaccines in 2007. Both were introduced in the public sector through donations to the Ministry of Health (MOH) [30,31]. GSK donated 50,000 doses of its bivalent vaccine (Cervarix) to vaccinate young girls in Ibanda and Nakasongola districts [30]. A total of 417 primary schools and 69 health facilities in these districts participated in the demonstration project [32]. A school-based strategy was used in Ibanda district to vaccinate all girls in primary 5. An age-based strategy was used in Nakasongola district to vaccinate girls aged 10 years on Child Days Plus (CDP); a program that targets all children aged ≤14 years with an integrated package of preventive services including catch-up immunizations [33,34]. Approximately 10,000 girls were fully vaccinated over a three-year period. Coverage rates were higher in Ibanda (>85%) than in Nakasongola (~50%) district primarily because of the criteria used to select the girls for vaccination [32].

Merck & Co., Inc. through the Gardasil Access Program donated 1,600 doses of Gardasil to vaccinate 500 HIV positive girls aged 9–15 years [31]. By the end of 2011, about 200 young girls had received HPV vaccination [35].

### Sustainable financing can be secured

The main sources of funding for the health sector include the central government, donor projects, global initiatives and households through user fees levied in the private wings of hospitals. Uganda is one of the world’s poorest countries with approximately 38.0% of her population living below the international poverty line [36]. Compared to USA, Uganda’s expenditure as percentage of GDP is low, 16.2% and 8.2%, respectively [37]. Less than 10% of the total annual budget is allocated to the health sector, which is far below the Abuja Declaration target of 15% [38,39]. Uganda’s per capita expenditure on health estimated at only US$12.5 falls short of the US$34 per capita recommended by the WHO for delivery of a basic health care package that includes immunization [40].

### The cost-effectiveness of vaccination strategies in the country or region is considered

Cost-effective analysis of HPV 16/18 vaccination in the 72 GAVI-eligible countries including Uganda has recently been published [41]. Compared to no vaccination, at a cost of US$2 per vaccine dose, which is equivalent to International dollar (I$)10 per vaccinated adolescent girl, HPV vaccination would be cost-effective in Uganda (Table 1). Further, with optimal (≥70%) vaccine coverage of a cohort of adolescent girls, the mean lifetime risk of cervical cancer for Ugandan women could be reduced by more than 50% [41]. 

**Table 1:** Cost effective analysis of vaccinating a single cohort of 12 year old girls with HPV 16/18 vaccine for 5 East African Countries

**Country**	**Reduction in lifetime risk of cervical cancer (%)**	**Cancer Incidence (ASR)**	**ICER (I$ DALY averted)**	**ICER (I$ DALY averted)**	**ICER (I$ DALY averted)**	**ICER (I$ DALY averted)**
				**[I$ 10 per vaccinated girl]**	**[I$ 25 per vaccinated girl]**	**[I$ 50 per vaccinated girl]**
Burundi	55.4	42.7	7,283	70	220	480
Kenya	55.4	28.7	18,047	60	320	770
Rwanda	55.4	49.4	8,786	^*c*^	130	370
Tanzania	50.7	68.6	50,110	50	160	340
Uganda	53.7	36.3	33,813	^*c*^	100	340

Source: Adopted from [41].

^*c*^ Strategy cost effective compared to no vaccination.

*ASR*: Age standardized incidence rate.

*DALYs*: Disability Adjusted Life Years.

*ICER*: Incremental cost-effectiveness ratios.

*I$*: International Dollar.

### HPV vaccination is targeted to adolescent girls prior to sexual debut

Uganda has one of the youngest populations in the world with 50% of her population aged below 15 years [42]. Although the median age of sexual debut for girls is approximately 17.5 years, 11.1% of girls have initiated sexual activity by the age of 15 years [43] A recent multi-site survey showed that 8% of Ugandan girls aged 12–14 years reported having had sexual intercourse [44]. Another survey conducted in 24 districts of Uganda showed that 6% girls aged 10–14 years reported a history of sexually transmitted infections [45]. These studies suggest the urgency of targeting young adolescent girls prior to exposure to the virus.

## Opportunities

Uganda has opportunities that could enhance the introduction of universal routine HPV vaccination of young adolescent girls including:

### Political commitment

The GOU is committed to protecting women of child bearing age from vaccine preventable diseases. In this regard, the budget line for vaccines and related supplies is protected in the MOH budget. Also, the ruling National Resistance Movement party in its manifesto 2011–2016 pledged to scale up HPV vaccination [46].

### Enabling laws and favourable policy environment

The Public Health Act 1935 (Ch 281) and 2000 revision and the Local Government Act (Ch 243) [47,48] mandate the GOU to purchase and introduce vaccines for the public good. In the health sector, the current policies on Health and Non Communicable Diseases [49] provide a framework for strengthening health systems in order to improve access to primary prevention measures. Such laws and policies would aid the introduction and sustenance of universal routine HPV vaccination program.

### Vaccine acceptability

The ultimate success of universal routine HPV vaccination will partly depend on acceptability of the HPV vaccine by the providers, parents and adolescents themselves. Although data on acceptability of the HPV vaccine is limited, health workers and teachers endorsed the HPV vaccine [30]. Additionally, acceptability by parents was high (80%) [30]. Parents were motivated to have their daughters vaccinated by their positive perception of the role of vaccines in disease prevention in general and that HPV vaccination was a government program [32]. The fact that approximately 10,000 girls completed all the 3 doses of vaccination demonstrated their acceptability of the HPV vaccine [30]. The reasons for non acceptance were more often driven by programmatic considerations e.g. school absenteeism on the day of vaccination rather than opposition to the vaccine [32].

### Partnerships

From past experience, support from partners during the initial phases of new vaccine introduction is critical. GAVI has recently offered new support for HPV vaccine [50]. Uganda has an opportunity to apply for the initial funding support from GAVI and then assume the responsibility for financing gradually.

### Creating synergies with existing programs

There is an opportunity to build synergies between HPV vaccination and existing health programs such as the bi-annual CDP program, which is widely accepted by the community [34] and the school health program for Tetanus Toxoid (TT) vaccination [51].

HPV vaccination will bring together different stakeholders including teachers, and health workers in the departments of reproductive health, immunization, adolescent health and cancer who normally do not work together. This provides an opportunity to pool together available resources. With ample planning and appropriate packaging of messages to the community, it is feasible to deliver an HPV vaccine to young adolescent girls using existing health and educational infrastructure if adequate financial resources are available [30].

## Potential obstacles

### Cost

The cost of the vaccine and its delivery is still a major potential obstacle. With Uganda’s high estimated population growth rate of 3.6% per annum [52], the growth of the immunization budget in real terms reduces significantly. This means that any increase in funding resources should be over and above the increase in the annual age cohort(s) targeted for HPV vaccination. What’s more, is the volatile foreign exchange rate. Should Uganda obtain GAVI funding support, issues of co-financing and financing of the HPV vaccination program beyond GAVI funding remains a major potential obstacle [53]. Without compulsory health insurance and with the low coverage of private health insurance, Uganda has limited pooling of resources, and hence minimal cross-subsidization [54]. In a recent study, the cost of providing a single dose of HPV vaccine (excluding cost of vaccine) through a vertical school-based vaccination strategy was US$3.15 [30] comparable to the cost of delivery (US$3.75) in Tanzania using the same strategy [55]. Conversely, the cost of delivering a dose of the HPV vaccine integrated into an existing outreach program was estimated at US$1.65 [30]. Overall, approximately US$4 to US$10 per fully vaccinated girl would be required for vaccine delivery costs.

### Cultural barriers

Cultural barriers may arise for a vaccine targeting only girls. From past experience, roumours that oral polio and TT vaccines in Uganda were actually an anti-fertility vaccines became widespread and were difficult to manage for some years [56]. Already, some few parents in communities that participated in one of the HPV demonstration projects were concerned about future fertility of the vaccinated girls [32]. Such issues underscore the importance of appropriate information and communication at all levels in the communities.

### Operational and logistical challenges

Although school attendance of girls who participated in the HPV demonstration project was high, attendance for girls in upper primary classes (P5-7) in general is unacceptably low as a result of high dropout rates and abseentism associated with menstruation [32,57]. In this regard, any delivery strategy that is used must consider ways of reaching girls who are absent on vaccination days or those who are out of school [32]. Given the widespread cultural practice of early marriages to older and sexually experienced partners in most Ugandan communities, out- of- school girls are the most vulnerable to the acquisition of HPV infection. Emphasis should be put on strategies such as integrating school-based and outreach strategy on CDP so as to reach as many adolescent girls with the HPV vaccine [30].

HPV vaccination will have an impact on the cold chain, frequency of re-supply and transport at different levels. With the existing infrastructure, the capacity to store HPV vaccines seems inadequate as a result of broken down equipment and shortage of gas cylinders required to run the refrigerators during frequent power outages [33]. Such challenges have been exacerbated by increase in the number of districts from 50 in 2004 to the current 112 where health infrastructure may be limited or nonexistent.

Introduction of routine HPV vaccination will bring together staff from the traditional infant and childhood immunization, sexual and reproductive health and cancer control. These stakeholders will be involved in decision making on issues related to immunization. There are currently on-going discussions within the MOH to introduce pneumococcal, rotavirus and HPV vaccines before 2015. These vaccines are more expensive than the traditional infant and childhood vaccines [58]. At a glance, it would seem reasonable to prioritize vaccines against childhood infections because they are associated with high mortality rates. However, preventing cervical cancer would save the lives of relatively young women who have critical roles in caring for children and their families thus maintaining stability of households and communities at large [59].

### Education of policy makers and the general public about HPV vaccines

Not all health policy makers are experts in the field of cervical cancer prevention and therefore may not be knowledgeable about HPV vaccines. Additionally, community members are generally uninformed about the need for vaccination of adolescents. An additional challenge would be how to provide culturally appropriate information to community members with limited or no reading skills, and who may not have access to regular mass media or may not even trust the government or health authorities [60]. Therefore, it will be critical for the MOH to involve local leaders in mobilization and outreach activities [30] and in addition partner with community- based public and private organization and their networks for delivery of effective community education.

## Conclusions

Uganda fulfils 4 out of the 5 criteria set by the WHO for the introduction of routine HPV vaccination to young adolescent girls. Given the high incidence of cervical cancer and in the absence of a national screening program, universal HPV vaccination of Ugandan adolescent girls is critical for cervical cancer prevention.

## Competing interests

The authors declare that they have no competing interests.

## Authors’ contributions

CB conceived the study, searched the literature, drafted the manuscript and produced the final table. FMM, ARK, PBN & EKM made substantial contributions to the manuscript and contributed to data interpretation. All authors read and approved the final manuscript.

## Author details

^1^Child Health and Development Centre, Makerere University College of Health Sciences, P. O. Box 6717, Kampala, Uganda. ^2^Department of Obstetrics and Gynaecology, Makerere University College of Health Sciences, P.O. Box 7072, Kampala, Uganda. ^3^Uganda Virus Research Institute, P.O. Box 49, Entebbe, Uganda. ^4^National Institute for Health and Welfare, Oulu, Finland.
